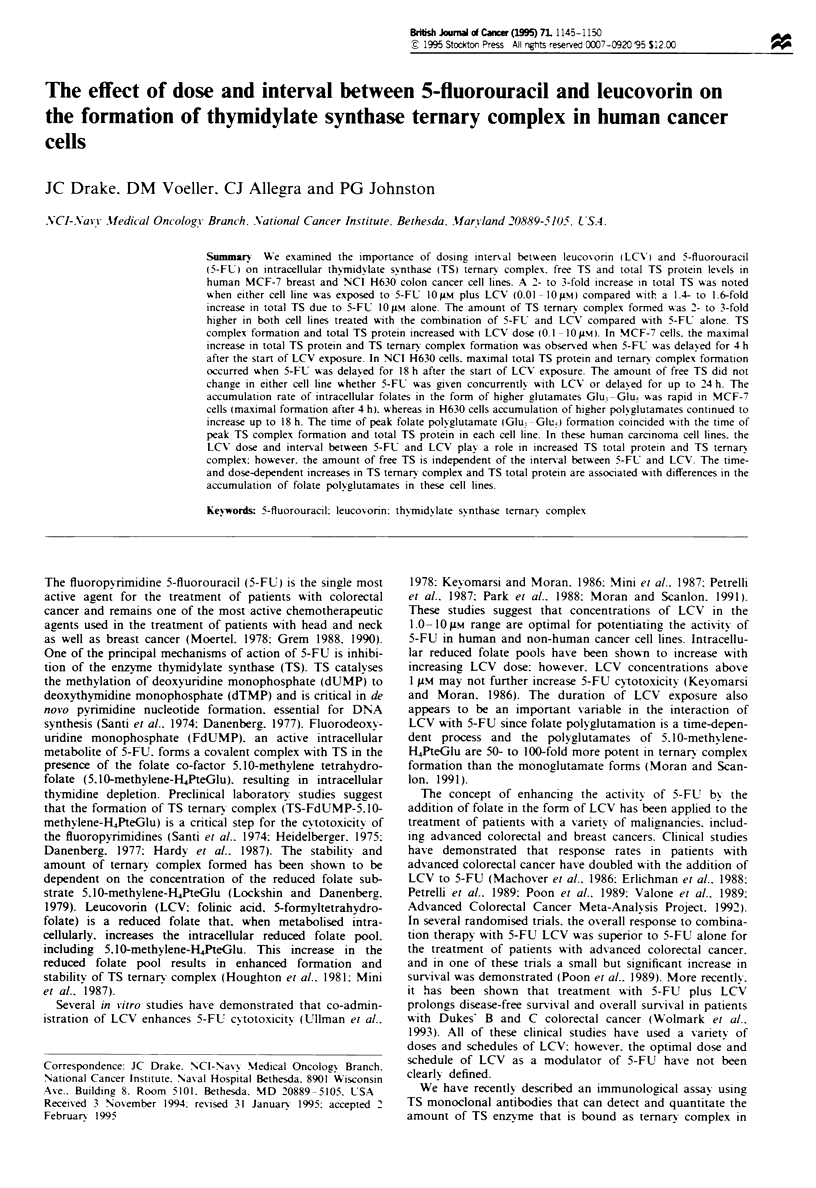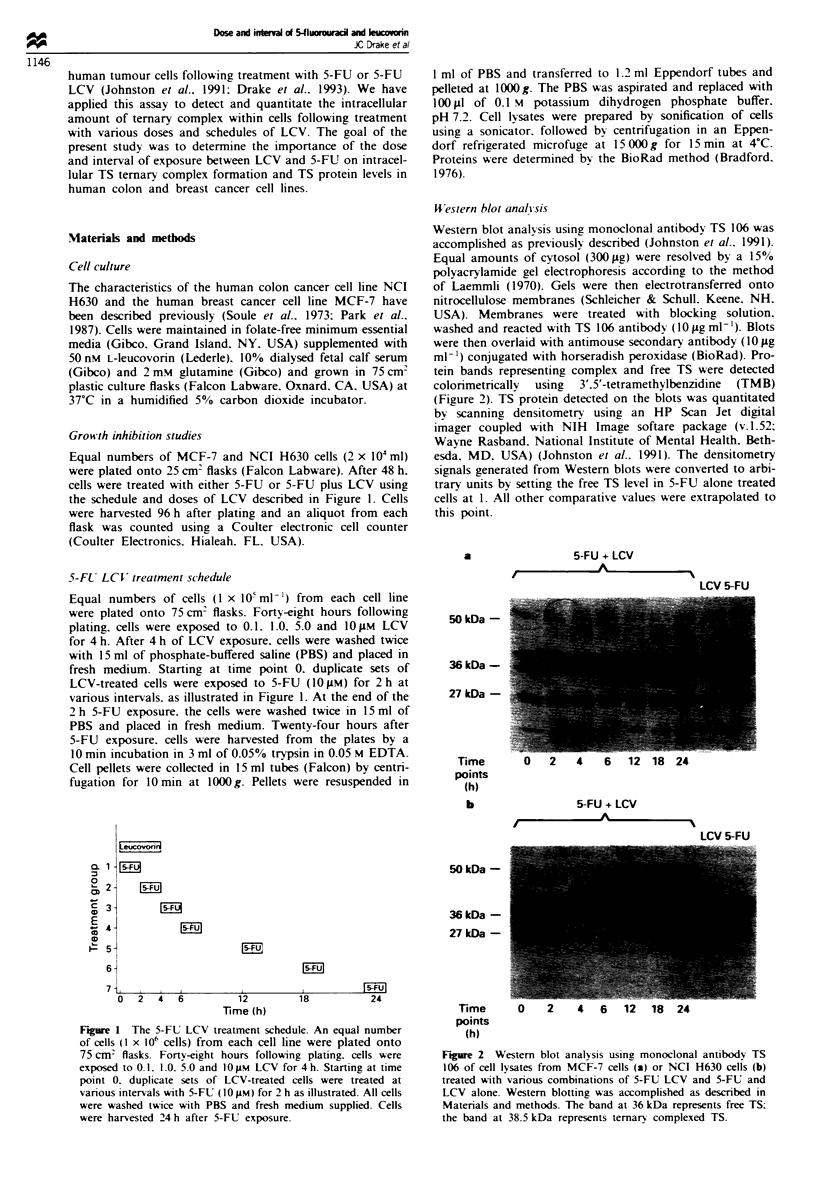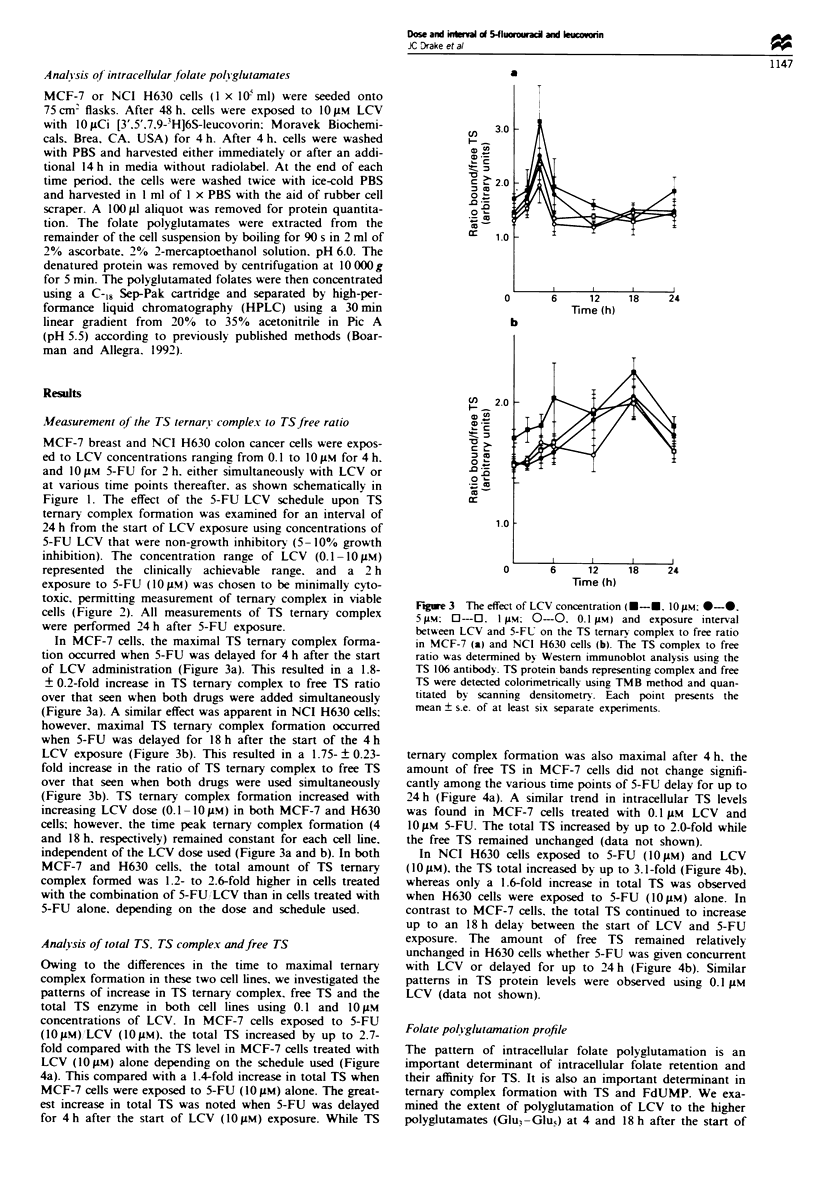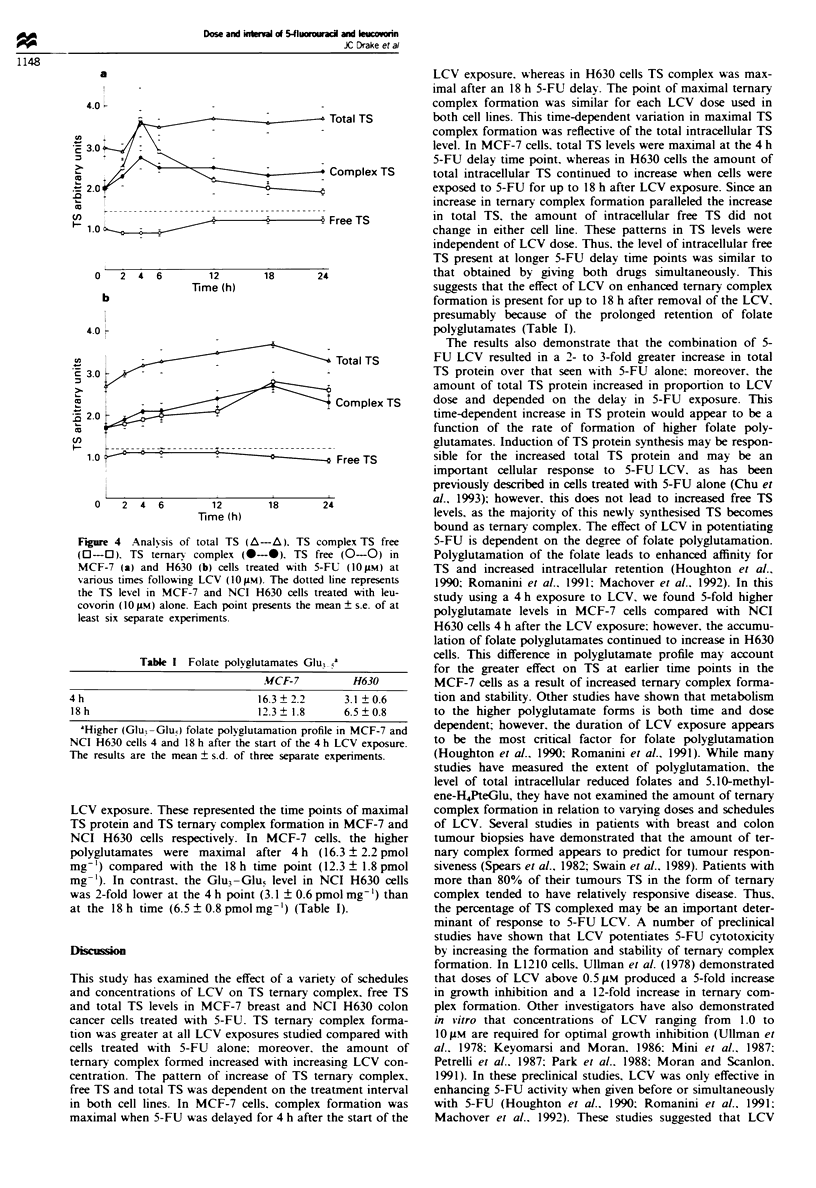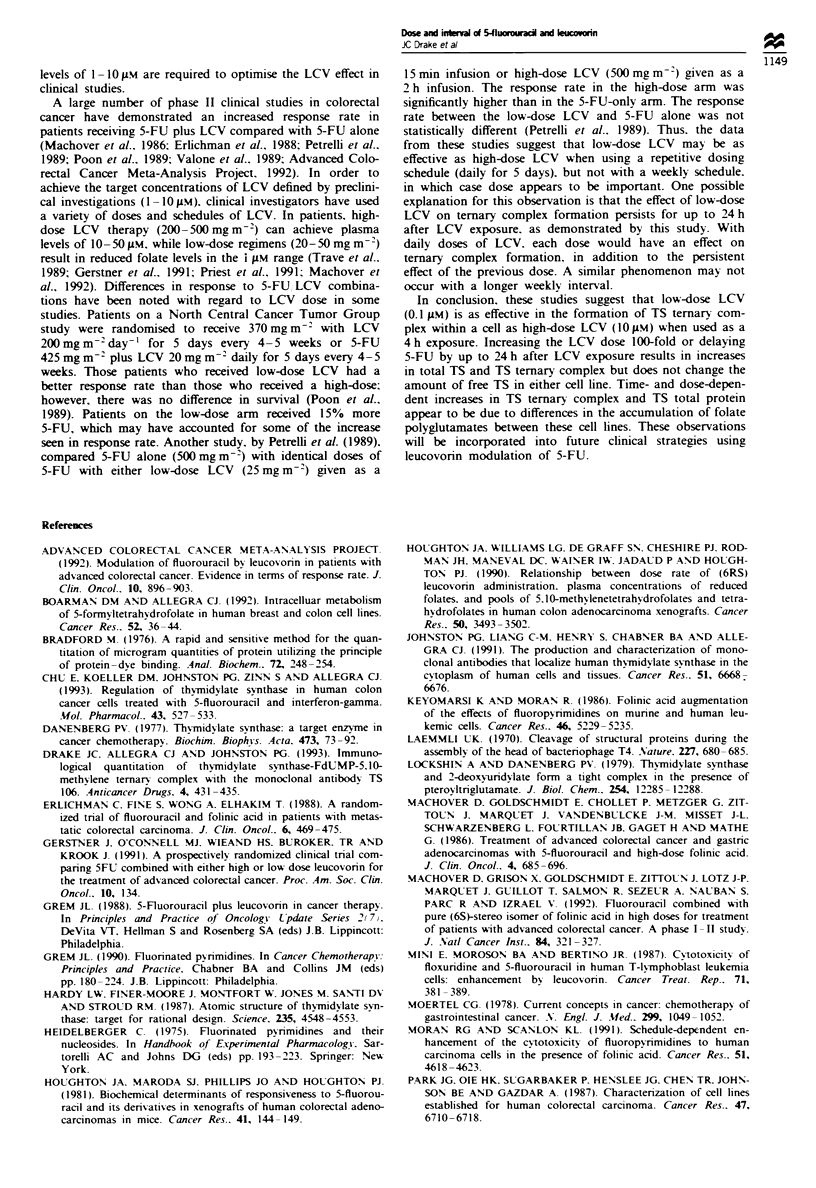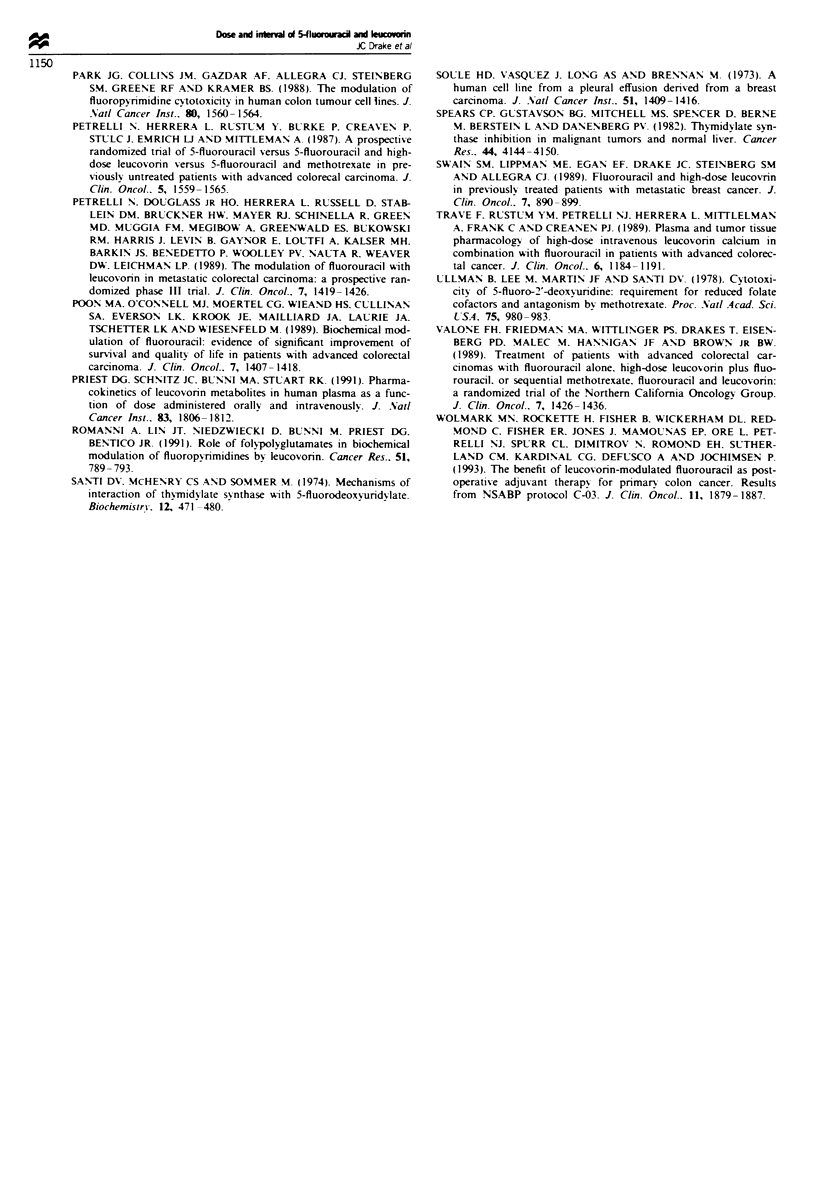# The effect of dose and interval between 5-fluorouracil and leucovorin on the formation of thymidylate synthase ternary complex in human cancer cells.

**DOI:** 10.1038/bjc.1995.224

**Published:** 1995-06

**Authors:** J. C. Drake, D. M. Voeller, C. J. Allegra, P. G. Johnston

**Affiliations:** NCI-Navy Medical Oncology Branch, Bethesda, Maryland 20889-5105, USA.

## Abstract

**Images:**


British Journal of Cancer (1995) 71, 1145–1150

© 1995 Stockton Press All rights reserved 0007-0920/95 $12.00

### The effect of dose and interval between 5-fluorouracil and leucovorin on the formation of thymidylate synthase ternary complex in human cancer cells

JC Drake, DM Voeller, CJ Allegra and PG Johnston

*NCI-Navy Medical Oncology Branch, National Cancer Institute, Bethesda, Maryland 20889-5105, USA.*

**Summary** We examined the importance of dosing interval between leucovorin (LCV) and 5-fluorouracil (5-FU) on intracellular thymidylate synthase (TS) ternary complex, free TS and total TS protein levels in human MCF-7 breast and NCI H630 colon cancer cell lines. A 2- to 3-fold increase in total TS was noted when either cell line was exposed to 5-FU 10 μM plus LCV (0.01–10 μM) compared with a 1.4- to 1.6-fold increase in total TS due to 5-FU 10 μM alone. The amount of TS ternary complex formed was 2- to 3-fold higher in both cell lines treated with the combination of 5-FU and LCV compared with 5-FU alone. TS complex formation and total TS protein increased with LCV dose (0.1–10 μM). In MCF-7 cells, the maximal increase in total TS protein and TS ternary complex formation was observed when 5-FU was delayed for 4 h after the start of LCV exposure. In NCI H630 cells, maximal total TS protein and ternary complex formation occurred when 5-FU was delayed for 18 h after the start of LCV exposure. The amount of free TS did not change in either cell line whether 5-FU was given concurrently with LCV or delayed for up to 24 h. The accumulation rate of intracellular folates in the form of higher glutamates $Glu_3$–$Glu_5$ was rapid in MCF-7 cells (maximal formation after 4 h), whereas in H630 cells accumulation of higher polyglutamates continued to increase up to 18 h. The time of peak folate polyglutamate ($Glu_3$–$Glu_5$) formation coincided with the time of peak TS complex formation and total TS protein in each cell line. In these human carcinoma cell lines, the LCV dose and interval between 5-FU and LCV play a role in increased TS total protein and TS ternary complex; however, the amount of free TS is independent of the interval between 5-FU and LCV. The time- and dose-dependent increases in TS ternary complex and TS total protein are associated with differences in the accumulation of folate polyglutamates in these cell lines.

**Keywords:** 5-fluorouracil; leucovorin; thymidylate synthase ternary complex

The fluoropyrimidine 5-fluorouracil (5-FU) is the single most active agent for the treatment of patients with colorectal cancer and remains one of the most active chemotherapeutic agents used in the treatment of patients with head and neck as well as breast cancer (Moertel, 1978; Grem 1988, 1990). One of the principal mechanisms of action of 5-FU is inhibition of the enzyme thymidylate synthase (TS). TS catalyses the methylation of deoxyuridine monophosphate (dUMP) to deoxythymidine monophosphate (dTMP) and is critical in *de novo* pyrimidine nucleotide formation, essential for DNA synthesis (Santi *et al.*, 1974; Danenberg, 1977). Fluorodeoxyuridine monophosphate (FdUMP), an active intracellular metabolite of 5-FU, forms a covalent complex with TS in the presence of the folate co-factor 5,10-methylene tetrahydrofolate (5,10-methylene-$H_4$PteGlu), resulting in intracellular thymidine depletion. Preclinical laboratory studies suggest that the formation of TS ternary complex (TS-FdUMP-5,10-methylene-$H_4$PteGlu) is a critical step for the cytotoxicity of the fluoropyrimidines (Santi *et al.*, 1974; Heidelberger, 1975; Danenberg, 1977; Hardy *et al.*, 1987). The stability and amount of ternary complex formed has been shown to be dependent on the concentration of the reduced folate substrate 5,10-methylene-$H_4$PteGlu (Lockshin and Danenberg, 1979). Leucovorin (LCV; folinic acid, 5-formyltetrahydrofolate) is a reduced folate that, when metabolised intracellularly, increases the intracellular reduced folate pool, including 5,10-methylene-$H_4$PteGlu. This increase in the reduced folate pool results in enhanced formation and stability of TS ternary complex (Houghton *et al.*, 1981; Mini *et al.*, 1987).

Several *in vitro* studies have demonstrated that co-administration of LCV enhances 5-FU cytotoxicity (Ullman *et al.*, 1978; Keyomarsi and Moran, 1986; Mini *et al.*, 1987; Petrelli *et al.*, 1987; Park *et al.*, 1988; Moran and Scanlon, 1991). These studies suggest that concentrations of LCV in the 1.0–10 μM range are optimal for potentiating the activity of 5-FU in human and non-human cancer cell lines. Intracellular reduced folate pools have been shown to increase with increasing LCV dose; however, LCV concentrations above 1 μM may not further increase 5-FU cytotoxicity (Keyomarsi and Moran, 1986). The duration of LCV exposure also appears to be an important variable in the interaction of LCV with 5-FU since folate polyglutamation is a time-dependent process and the polyglutamates of 5,10-methylene-$H_4$PteGlu are 50- to 100-fold more potent in ternary complex formation than the monoglutamate forms (Moran and Scanlon, 1991).

The concept of enhancing the activity of 5-FU by the addition of folate in the form of LCV has been applied to the treatment of patients with a variety of malignancies, including advanced colorectal and breast cancers. Clinical studies have demonstrated that response rates in patients with advanced colorectal cancer have doubled with the addition of LCV to 5-FU (Machover *et al.*, 1986; Erlichman *et al.*, 1988; Petrelli *et al.*, 1989; Poon *et al.*, 1989; Valone *et al.*, 1989; Advanced Colorectal Cancer Meta-Analysis Project, 1992). In several randomised trials, the overall response to combination therapy with 5-FU LCV was superior to 5-FU alone for the treatment of patients with advanced colorectal cancer, and in one of these trials a small but significant increase in survival was demonstrated (Poon *et al.*, 1989). More recently, it has been shown that treatment with 5-FU plus LCV prolongs disease-free survival and overall survival in patients with Dukes' B and C colorectal cancer (Wolmark *et al.*, 1993). All of these clinical studies have used a variety of doses and schedules of LCV; however, the optimal dose and schedule of LCV as a modulator of 5-FU have not been clearly defined.

We have recently described an immunological assay using TS monoclonal antibodies that can detect and quantitate the amount of TS enzyme that is bound as ternary complex in

Correspondence: JC Drake, NCI-Navy Medical Oncology Branch, National Cancer Institute, Naval Hospital Bethesda, 8901 Wisconsin Ave., Building 8, Room 5101, Bethesda, MD 20889-5105, USA
Received 3 November 1994; revised 31 January 1995; accepted 2 February 1995

human tumour cells following treatment with 5-FU or 5-FU LCV (Johnston *et al*., 1991; Drake *et al*., 1993). We have applied this assay to detect and quantitate the intracellular amount of ternary complex within cells following treatment with various doses and schedules of LCV. The goal of the present study was to determine the importance of the dose and interval of exposure between LCV and 5-FU on intracellular TS ternary complex formation and TS protein levels in human colon and breast cancer cell lines.

### Materials and methods

### *Cell culture*

The characteristics of the human colon cancer cell line NCI H630 and the human breast cancer cell line MCF-7 have been described previously (Soule *et al*., 1973; Park *et al*., 1987). Cells were maintained in folate-free minimum essential media (Gibco, Grand Island, NY, USA) supplemented with 50 nM L-leucovorin (Lederle), 10% dialysed fetal calf serum (Gibco) and 2 mM glutamine (Gibco) and grown in 75 $cm^2$ plastic culture flasks (Falcon Labware, Oxnard, CA, USA) at 37°C in a humidified 5% carbon dioxide incubator.

### *Growth inhibition studies*

Equal numbers of MCF-7 and NCI H630 cells ($2 \times 10^4$ ml) were plated onto 25 $cm^2$ flasks (Falcon Labware). After 48 h, cells were treated with either 5-FU or 5-FU plus LCV using the schedule and doses of LCV described in Figure 1. Cells were harvested 96 h after plating and an aliquot from each flask was counted using a Coulter electronic cell counter (Coulter Electronics, Hialeah, FL, USA).

### *5-FU LCV treatment schedule*

Equal numbers of cells ($1 \times 10^5$ $ml^{-1}$) from each cell line were plated onto 75 $cm^2$ flasks. Forty-eight hours following plating, cells were exposed to 0.1, 1.0, 5.0 and 10 µM LCV for 4 h. After 4 h of LCV exposure, cells were washed twice with 15 ml of phosphate-buffered saline (PBS) and placed in fresh medium. Starting at time point 0, duplicate sets of LCV-treated cells were exposed to 5-FU (10 µM) for 2 h at various intervals, as illustrated in Figure 1. At the end of the 2 h 5-FU exposure, the cells were washed twice in 15 ml of PBS and placed in fresh medium. Twenty-four hours after 5-FU exposure, cells were harvested from the plates by a 10 min incubation in 3 ml of 0.05% trypsin in 0.05 M EDTA. Cell pellets were collected in 15 ml tubes (Falcon) by centrifugation for 10 min at 1000 *g*. Pellets were resuspended in 1 ml of PBS and transferred to 1.2 ml Eppendorf tubes and pelleted at 1000 *g*. The PBS was aspirated and replaced with 100 µl of 0.1 M potassium dihydrogen phosphate buffer, pH 7.2. Cell lysates were prepared by sonification of cells using a sonicator, followed by centrifugation in an Eppendorf refrigerated microfuge at 15 000 *g* for 15 min at 4°C. Proteins were determined by the BioRad method (Bradford, 1976).

**Figure 1** The 5-FU LCV treatment schedule. An equal number of cells ($1 \times 10^6$ cells) from each cell line were plated onto 75 $cm^2$ flasks. Forty-eight hours following plating, cells were exposed to 0.1, 1.0, 5.0 and 10 µM LCV for 4 h. Starting at time point 0, duplicate sets of LCV-treated cells were treated at various intervals with 5-FU (10 µM) for 2 h as illustrated. All cells were washed twice with PBS and fresh medium supplied. Cells were harvested 24 h after 5-FU exposure.

### *Western blot analysis*

Western blot analysis using monoclonal antibody TS 106 was accomplished as previously described (Johnston *et al*., 1991). Equal amounts of cytosol (300 µg) were resolved by a 15% polyacrylamide gel electrophoresis according to the method of Laemmli (1970). Gels were then electrotransferred onto nitrocellulose membranes (Schleicher & Schull, Keene, NH, USA). Membranes were treated with blocking solution, washed and reacted with TS 106 antibody (10 µg $ml^{-1}$). Blots were then overlaid with antimouse secondary antibody (10 µg $ml^{-1}$) conjugated with horseradish peroxidase (BioRad). Protein bands representing complex and free TS were detected colorimetrically using 3′,5′-tetramethylbenzidine (TMB) (Figure 2). TS protein detected on the blots was quantitated by scanning densitometry using an HP Scan Jet digital imager coupled with NIH Image softare package (v.1.52; Wayne Rasband, National Institute of Mental Health, Bethesda, MD, USA) (Johnston *et al*., 1991). The densitometry signals generated from Western blots were converted to arbitrary units by setting the free TS level in 5-FU alone treated cells at 1. All other comparative values were extrapolated to this point.

**Figure 2** Western blot analysis using monoclonal antibody TS 106 of cell lysates from MCF-7 cells (**a**) or NCI H630 cells (**b**) treated with various combinations of 5-FU LCV and 5-FU and LCV alone. Western blotting was accomplished as described in Materials and methods. The band at 36 kDa represents free TS; the band at 38.5 kDa represents ternary complexed TS.

### Analysis of intracellular folate polyglutamates

MCF-7 or NCI H630 cells ($1 \times 10^5$ ml) were seeded onto 75 $cm^2$ flasks. After 48 h, cells were exposed to 10 μM LCV with 10 μCi [3',5',7,9-$^3$H]6S-leucovorin; Moravek Biochemicals, Brea, CA, USA) for 4 h. After 4 h, cells were washed with PBS and harvested either immediately or after an additional 14 h in media without radiolabel. At the end of each time period, the cells were washed twice with ice-cold PBS and harvested in 1 ml of 1 × PBS with the aid of rubber cell scraper. A 100 μl aliquot was removed for protein quantitation. The folate polyglutamates were extracted from the remainder of the cell suspension by boiling for 90 s in 2 ml of 2% ascorbate, 2% 2-mercaptoethanol solution, pH 6.0. The denatured protein was removed by centrifugation at 10 000 *g* for 5 min. The polyglutamated folates were then concentrated using a $C_{18}$ Sep-Pak cartridge and separated by high-performance liquid chromatography (HPLC) using a 30 min linear gradient from 20% to 35% acetonitrile in Pic A (pH 5.5) according to previously published methods (Boarman and Allegra, 1992).

### Results

### Measurement of the TS ternary complex to TS free ratio

MCF-7 breast and NCI H630 colon cancer cells were exposed to LCV concentrations ranging from 0.1 to 10 μM for 4 h, and 10 μM 5-FU for 2 h, either simultaneously with LCV or at various time points thereafter, as shown schematically in Figure 1. The effect of the 5-FU LCV schedule upon TS ternary complex formation was examined for an interval of 24 h from the start of LCV exposure using concentrations of 5-FU LCV that were non-growth inhibitory (5–10% growth inhibition). The concentration range of LCV (0.1–10 μM) represented the clinically achievable range, and a 2 h exposure to 5-FU (10 μM) was chosen to be minimally cytotoxic, permitting measurement of ternary complex in viable cells (Figure 2). All measurements of TS ternary complex were performed 24 h after 5-FU exposure.

In MCF-7 cells, the maximal TS ternary complex formation occurred when 5-FU was delayed for 4 h after the start of LCV administration (Figure 3a). This resulted in a 1.8- ± 0.2-fold increase in TS ternary complex to free TS ratio over that seen when both drugs were added simultaneously (Figure 3a). A similar effect was apparent in NCI H630 cells; however, maximal TS ternary complex formation occurred when 5-FU was delayed for 18 h after the start of the 4 h LCV exposure (Figure 3b). This resulted in a 1.75- ± 0.23-fold increase in the ratio of TS ternary complex to free TS over that seen when both drugs were used simultaneously (Figure 3b). TS ternary complex formation increased with increasing LCV dose (0.1–10 μM) in both MCF-7 and H630 cells; however, the time peak ternary complex formation (4 and 18 h, respectively) remained constant for each cell line, independent of the LCV dose used (Figure 3a and b). In both MCF-7 and H630 cells, the total amount of TS ternary complex formed was 1.2- to 2.6-fold higher in cells treated with the combination of 5-FU/LCV than in cells treated with 5-FU alone, depending on the dose and schedule used.

### Analysis of total TS, TS complex and free TS

Owing to the differences in the time to maximal ternary complex formation in these two cell lines, we investigated the patterns of increase in TS ternary complex, free TS and the total TS enzyme in both cell lines using 0.1 and 10 μM concentrations of LCV. In MCF-7 cells exposed to 5-FU (10 μM)/LCV (10 μM), the total TS increased by up to 2.7-fold compared with the TS level in MCF-7 cells treated with LCV (10 μM) alone depending on the schedule used (Figure 4a). This compared with a 1.4-fold increase in total TS when MCF-7 cells were exposed to 5-FU (10 μM) alone. The greatest increase in total TS was noted when 5-FU was delayed for 4 h after the start of LCV (10 μM) exposure. While TS

**Figure 3** The effect of LCV concentration (■—■, 10 μM; ●—●, 5 μM; □—□, 1 μM; ○—○, 0.1 μM) and exposure interval between LCV and 5-FU on the TS ternary complex to free ratio in MCF-7 (**a**) and NCI H630 cells (**b**). The TS complex to free ratio was determined by Western immunoblot analysis using the TS 106 antibody. TS protein bands representing complex and free TS were detected colorimetrically using TMB method and quantitated by scanning densitometry. Each point presents the mean ± s.e. of at least six separate experiments.

ternary complex formation was also maximal after 4 h, the amount of free TS in MCF-7 cells did not change significantly among the various time points of 5-FU delay for up to 24 h (Figure 4a). A similar trend in intracellular TS levels was found in MCF-7 cells treated with 0.1 μM LCV and 10 μM 5-FU. The total TS increased by up to 2.0-fold while the free TS remained unchanged (data not shown).

In NCI H630 cells exposed to 5-FU (10 μM) and LCV (10 μM), the TS total increased by up to 3.1-fold (Figure 4b), whereas only a 1.6-fold increase in total TS was observed when H630 cells were exposed to 5-FU (10 μM) alone. In contrast to MCF-7 cells, the total TS continued to increase up to an 18 h delay between the start of LCV and 5-FU exposure. The amount of free TS remained relatively unchanged in H630 cells whether 5-FU was given concurrent with LCV or delayed for up to 24 h (Figure 4b). Similar patterns in TS protein levels were observed using 0.1 μM LCV (data not shown).

### Folate polyglutamation profile

The pattern of intracellular folate polyglutamation is an important determinant of intracellular folate retention and their affinity for TS. It is also an important determinant in ternary complex formation with TS and FdUMP. We examined the extent of polyglutamation of LCV to the higher polyglutamates ($Glu_3$–$Glu_5$) at 4 and 18 h after the start of

**Figure 4** Analysis of total TS (Δ—Δ), TS complex TS free (□—□), TS ternary complex (●—●), TS free (O—O) in MCF-7 (**a**) and H630 (**b**) cells treated with 5-FU (10 µM) at various times following LCV (10 µM). The dotted line represents the TS level in MCF-7 and NCI H630 cells treated with leucovorin (10 µM) alone. Each point presents the mean ± s.e. of at least six separate experiments.

**Table I** Folate polyglutamates $Glu_{3-5}$[a]

| | *MCF-7* | *H630* |
|---|---|---|
| 4 h | 16.3 ± 2.2 | 3.1 ± 0.6 |
| 18 h | 12.3 ± 1.8 | 6.5 ± 0.8 |

[a]Higher ($Glu_3$–$Glu_5$) folate polyglutamation profile in MCF-7 and NCI H630 cells 4 and 18 h after the start of the 4 h LCV exposure. The results are the mean ± s.d. of three separate experiments.

LCV exposure. These represented the time points of maximal TS protein and TS ternary complex formation in MCF-7 and NCI H630 cells respectively. In MCF-7 cells, the higher polyglutamates were maximal after 4 h (16.3 ± 2.2 pmol $mg^{-1}$) compared with the 18 h time point (12.3 ± 1.8 pmol $mg^{-1}$). In contrast, the $Glu_3$–$Glu_5$ level in NCI H630 cells was 2-fold lower at the 4 h point (3.1 ± 0.6 pmol $mg^{-1}$) than at the 18 h time (6.5 ± 0.8 pmol $mg^{-1}$) (Table I).

### Discussion

This study has examined the effect of a variety of schedules and concentrations of LCV on TS ternary complex, free TS and total TS levels in MCF-7 breast and NCI H630 colon cancer cells treated with 5-FU. TS ternary complex formation was greater at all LCV exposures studied compared with cells treated with 5-FU alone; moreover, the amount of ternary complex formed increased with increasing LCV concentration. The pattern of increase of TS ternary complex, free TS and total TS was dependent on the treatment interval in both cell lines. In MCF-7 cells, complex formation was maximal when 5-FU was delayed for 4 h after the start of the LCV exposure, whereas in H630 cells TS complex was maximal after an 18 h 5-FU delay. The point of maximal ternary complex formation was similar for each LCV dose used in both cell lines. This time-dependent variation in maximal TS complex formation was reflective of the total intracellular TS level. In MCF-7 cells, total TS levels were maximal at the 4 h 5-FU delay time point, whereas in H630 cells the amount of total intracellular TS continued to increase when cells were exposed to 5-FU for up to 18 h after LCV exposure. Since an increase in ternary complex formation paralleled the increase in total TS, the amount of intracellular free TS did not change in either cell line. These patterns in TS levels were independent of LCV dose. Thus, the level of intracellular free TS present at longer 5-FU delay time points was similar to that obtained by giving both drugs simultaneously. This suggests that the effect of LCV on enhanced ternary complex formation is present for up to 18 h after removal of the LCV, presumably because of the prolonged retention of folate polyglutamates (Table I).

The results also demonstrate that the combination of 5-FU LCV resulted in a 2- to 3-fold greater increase in total TS protein over that seen with 5-FU alone; moreover, the amount of total TS protein increased in proportion to LCV dose and depended on the delay in 5-FU exposure. This time-dependent increase in TS protein would appear to be a function of the rate of formation of higher folate polyglutamates. Induction of TS protein synthesis may be responsible for the increased total TS protein and may be an important cellular response to 5-FU LCV, as has been previously described in cells treated with 5-FU alone (Chu *et al.*, 1993); however, this does not lead to increased free TS levels, as the majority of this newly synthesised TS becomes bound as ternary complex. The effect of LCV in potentiating 5-FU is dependent on the degree of folate polyglutamation. Polyglutamation of the folate leads to enhanced affinity for TS and increased intracellular retention (Houghton *et al.*, 1990; Romanini *et al.*, 1991; Machover *et al.*, 1992). In this study using a 4 h exposure to LCV, we found 5-fold higher polyglutamate levels in MCF-7 cells compared with NCI H630 cells 4 h after the LCV exposure; however, the accumulation of folate polyglutamates continued to increase in H630 cells. This difference in polyglutamate profile may account for the greater effect on TS at earlier time points in the MCF-7 cells as a result of increased ternary complex formation and stability. Other studies have shown that metabolism to the higher polyglutamate forms is both time and dose dependent; however, the duration of LCV exposure appears to be the most critical factor for folate polyglutamation (Houghton *et al.*, 1990; Romanini *et al.*, 1991). While many studies have measured the extent of polyglutamation, the level of total intracellular reduced folates and 5,10-methylene-$H_4$PteGlu, they have not examined the amount of ternary complex formation in relation to varying doses and schedules of LCV. Several studies in patients with breast and colon tumour biopsies have demonstrated that the amount of ternary complex formed appears to predict for tumour responsiveness (Spears *et al.*, 1982; Swain *et al.*, 1989). Patients with more than 80% of their tumours TS in the form of ternary complex tended to have relatively responsive disease. Thus, the percentage of TS complexed may be an important determinant of response to 5-FU LCV. A number of preclinical studies have shown that LCV potentiates 5-FU cytotoxicity by increasing the formation and stability of ternary complex formation. In L1210 cells, Ullman *et al.* (1978) demonstrated that doses of LCV above 0.5 µM produced a 5-fold increase in growth inhibition and a 12-fold increase in ternary complex formation. Other investigators have also demonstrated *in vitro* that concentrations of LCV ranging from 1.0 to 10 µM are required for optimal growth inhibition (Ullman *et al.*, 1978; Keyomarsi and Moran, 1986; Mini *et al.*, 1987; Petrelli *et al.*, 1987; Park *et al.*, 1988; Moran and Scanlon, 1991). In these preclinical studies, LCV was only effective in enhancing 5-FU activity when given before or simultaneously with 5-FU (Houghton *et al.*, 1990; Romanini *et al.*, 1991; Machover *et al.*, 1992). These studies suggested that LCV

levels of 1–10 μM are required to optimise the LCV effect in clinical studies.

A large number of phase II clinical studies in colorectal cancer have demonstrated an increased response rate in patients receiving 5-FU plus LCV compared with 5-FU alone (Machover *et al*., 1986; Erlichman *et al*., 1988; Petrelli *et al*., 1989; Poon *et al*., 1989; Valone *et al*., 1989; Advanced Colorectal Cancer Meta-Analysis Project, 1992). In order to achieve the target concentrations of LCV defined by preclinical investigations (1–10 μM), clinical investigators have used a variety of doses and schedules of LCV. In patients, high-dose LCV therapy (200–500 mg $m^{-2}$) can achieve plasma levels of 10–50 μM, while low-dose regimens (20–50 mg $m^{-2}$) result in reduced folate levels in the 1 μM range (Trave *et al*., 1989; Gerstner *et al*., 1991; Priest *et al*., 1991; Machover *et al*., 1992). Differences in response to 5-FU/LCV combinations have been noted with regard to LCV dose in some studies. Patients on a North Central Cancer Tumor Group study were randomised to receive 370 mg $m^{-2}$ with LCV 200 mg $m^{-2}$ $day^{-1}$ for 5 days every 4–5 weeks or 5-FU 425 mg $m^{-2}$ plus LCV 20 mg $m^{-2}$ daily for 5 days every 4–5 weeks. Those patients who received low-dose LCV had a better response rate than those who received a high-dose; however, there was no difference in survival (Poon *et al*., 1989). Patients on the low-dose arm received 15% more 5-FU, which may have accounted for some of the increase seen in response rate. Another study, by Petrelli *et al*. (1989), compared 5-FU alone (500 mg $m^{-2}$) with identical doses of 5-FU with either low-dose LCV (25 mg $m^{-2}$) given as a 15 min infusion or high-dose LCV (500 mg $m^{-2}$) given as a 2 h infusion. The response rate in the high-dose arm was significantly higher than in the 5-FU-only arm. The response rate between the low-dose LCV and 5-FU alone was not statistically different (Petrelli *et al*., 1989). Thus, the data from these studies suggest that low-dose LCV may be as effective as high-dose LCV when using a repetitive dosing schedule (daily for 5 days), but not with a weekly schedule, in which case dose appears to be important. One possible explanation for this observation is that the effect of low-dose LCV on ternary complex formation persists for up to 24 h after LCV exposure, as demonstrated by this study. With daily doses of LCV, each dose would have an effect on ternary complex formation, in addition to the persistent effect of the previous dose. A similar phenomenon may not occur with a longer weekly interval.

In conclusion, these studies suggest that low-dose LCV (0.1 μM) is as effective in the formation of TS ternary complex within a cell as high-dose LCV (10 μM) when used as a 4 h exposure. Increasing the LCV dose 100-fold or delaying 5-FU by up to 24 h after LCV exposure results in increases in total TS and TS ternary complex but does not change the amount of free TS in either cell line. Time- and dose-dependent increases in TS ternary complex and TS total protein appear to be due to differences in the accumulation of folate polyglutamates between these cell lines. These observations will be incorporated into future clinical strategies using leucovorin modulation of 5-FU.

### References

ADVANCED COLORECTAL CANCER META-ANALYSIS PROJECT. (1992). Modulation of fluorouracil by leucovorin in patients with advanced colorectal cancer. Evidence in terms of response rate. *J. Clin. Oncol*., **10**, 896–903.

BOARMAN DM AND ALLEGRA CJ. (1992). Intracelluar metabolism of 5-formyltetrahydrofolate in human breast and colon cell lines. *Cancer Res*., **52**, 36–44.

BRADFORD M. (1976). A rapid and sensitive method for the quantitation of microgram quantities of protein utilizing the principle of protein-dye binding. *Anal. Biochem*., **72**, 248–254.

CHU E, KOELLER DM, JOHNSTON PG, ZINN S AND ALLEGRA CJ. (1993). Regulation of thymidylate synthase in human colon cancer cells treated with 5-fluorouracil and interferon-gamma. *Mol. Pharmacol*., **43**, 527–533.

DANENBERG PV. (1977). Thymidylate synthase: a target enzyme in cancer chemotherapy. *Biochim. Biophys. Acta*, **473**, 73–92.

DRAKE JC, ALLEGRA CJ AND JOHNSTON PG. (1993). Immunological quantitation of thymidylate synthase-FdUMP-5,10-methylene ternary complex with the monoclonal antibody TS 106. *Anticancer Drugs*, **4**, 431–435.

ERLICHMAN C, FINE S, WONG A, ELHAKIM T. (1988). A randomized trial of fluorouracil and folinic acid in patients with metastatic colorectal carcinoma. *J. Clin. Oncol*., **6**, 469–475.

GERSTNER J, O'CONNELL MJ, WIEAND HS, BUROKER, TR AND KROOK J. (1991). A prospectively randomized clinical trial comparing 5FU combined with either high or low dose leucovorin for the treatment of advanced colorectal cancer. *Proc. Am. Soc. Clin. Oncol*., **10**, 134.

GREM JL. (1988). 5-Fluorouracil plus leucovorin in cancer therapy. In *Principles and Practice of Oncology Update Series 2(7)*, DeVita VT, Hellman S and Rosenberg SA (eds) J.B. Lippincott: Philadelphia.

GREM JL. (1990). Fluorinated pyrimidines. In *Cancer Chemotherapy: Principles and Practice*, Chabner BA and Collins JM (eds) pp. 180–224. J.B. Lippincott: Philadelphia.

HARDY LW, FINER-MOORE J, MONTFORT W, JONES M, SANTI DV AND STROUD RM. (1987). Atomic structure of thymidylate synthase: target for rational design. *Science*, **235**, 4548–4553.

HEIDELBERGER C. (1975). Fluorinated pyrimidines and their nucleosides. In *Handbook of Experimental Pharmacology*, Sartorelli AC and Johns DG (eds) pp. 193–223. Springer: New York.

HOUGHTON JA, MARODA SJ, PHILLIPS JO AND HOUGHTON PJ. (1981). Biochemical determinants of responsiveness to 5-fluorouracil and its derivatives in xenografts of human colorectal adenocarcinomas in mice. *Cancer Res*., **41**, 144–149.

HOUGHTON JA, WILLIAMS LG, DE GRAFF SN, CHESHIRE PJ, RODMAN JH, MANEVAL DC, WAINER IW, JADAUD P AND HOUGHTON PJ. (1990). Relationship between dose rate of (6RS) leucovorin administration, plasma concentrations of reduced folates, and pools of 5,10-methylenetetrahydrofolates and tetrahydrofolates in human colon adenocarcinoma xenografts. *Cancer Res*., **50**, 3493–3502.

JOHNSTON PG, LIANG C-M, HENRY S, CHABNER BA AND ALLEGRA CJ. (1991). The production and characterization of monoclonal antibodies that localize human thymidylate synthase in the cytoplasm of human cells and tissues. *Cancer Res*., **51**, 6668–6676.

KEYOMARSI K AND MORAN R. (1986). Folinic acid augmentation of the effects of fluoropyrimidines on murine and human leukemic cells. *Cancer Res*., **46**, 5229–5235.

LAEMMLI UK. (1970). Cleavage of structural proteins during the assembly of the head of bacteriophage T4. *Nature*, **227**, 680–685.

LOCKSHIN A AND DANENBERG PV. (1979). Thymidylate synthase and 2-deoxyuridylate form a tight complex in the presence of pteroyltriglutamate. *J. Biol. Chem*., **254**, 12285–12288.

MACHOVER D, GOLDSCHMIDT E, CHOLLET P, METZGER G, ZITTOUN J, MARQUET J, VANDENBULCKE J-M, MISSET J-L, SCHWARZENBERG L, FOURTILLAN JB, GAGET H AND MATHE G. (1986). Treatment of advanced colorectal cancer and gastric adenocarcinomas with 5-fluorouracil and high-dose folinic acid. *J. Clin. Oncol*., **4**, 685–696.

MACHOVER D, GRISON X, GOLDSCHMIDT E, ZITTOUN J, LOTZ J-P, MARQUET J, GUILLOT T, SALMON R, SEZEUR A, NAUBAN S, PARC R AND IZRAEL V. (1992). Fluorouracil combined with pure (6S)-stereo isomer of folinic acid in high doses for treatment of patients with advanced colorectal cancer. A phase I–II study. *J. Natl Cancer Inst*., **84**, 321–327.

MINI E, MOROSON BA AND BERTINO JR. (1987). Cytotoxicity of floxuridine and 5-fluorouracil in human T-lymphoblast leukemia cells: enhancement by leucovorin. *Cancer Treat. Rep*., **71**, 381–389.

MOERTEL CG. (1978). Current concepts in cancer: chemotherapy of gastrointestinal cancer. *N. Engl. J. Med*., **299**, 1049–1052.

MORAN RG AND SCANLON KL. (1991). Schedule-dependent enhancement of the cytotoxicity of fluoropyrimidines to human carcinoma cells in the presence of folinic acid. *Cancer Res*., **51**, 4618–4623.

PARK JG, OIE HK, SUGARBAKER P, HENSLEE JG, CHEN TR, JOHNSON BE AND GAZDAR A. (1987). Characterization of cell lines established for human colorectal carcinoma. *Cancer Res*., **47**, 6710–6718.

PARK JG, COLLINS JM, GAZDAR AF, ALLEGRA CJ, STEINBERG SM, GREENE RF AND KRAMER BS. (1988). The modulation of fluoropyrimidine cytotoxicity in human colon tumour cell lines. *J. Natl Cancer Inst.*, **80,** 1560–1564.

PETRELLI N, HERRERA L, RUSTUM Y, BURKE P, CREAVEN P, STULC J, EMRICH LJ AND MITTLEMAN A. (1987). A prospective randomized trial of 5-fluorouracil versus 5-fluorouracil and high-dose leucovorin versus 5-fluorouracil and methotrexate in previously untreated patients with advanced colorecal carcinoma. *J. Clin. Oncol.*, **5,** 1559–1565.

PETRELLI N, DOUGLASS JR HO, HERRERA L, RUSSELL D, STABLEIN DM, BRUCKNER HW, MAYER RJ, SCHINELLA R, GREEN MD, MUGGIA FM, MEGIBOW A, GREENWALD ES, BUKOWSKI RM, HARRIS J, LEVIN B, GAYNOR E, LOUTFI A, KALSER MH, BARKIN JS, BENEDETTO P, WOOLLEY PV, NAUTA R, WEAVER DW, LEICHMAN LP. (1989). The modulation of fluorouracil with leucovorin in metastatic colorectal carcinoma: a prospective randomized phase III trial. *J. Clin. Oncol.*, **7,** 1419–1426.

POON MA, O'CONNELL MJ, MOERTEL CG, WIEAND HS, CULLINAN SA, EVERSON LK, KROOK JE, MAILLIARD JA, LAURIE JA, TSCHETTER LK AND WIESENFELD M. (1989). Biochemical modulation of fluorouracil: evidence of significant improvement of survival and quality of life in patients with advanced colorectal carcinoma. *J. Clin. Oncol.*, **7,** 1407–1418.

PRIEST DG, SCHNITZ JC, BUNNI MA, STUART RK. (1991). Pharmacokinetics of leucovorin metabolites in human plasma as a function of dose administered orally and intravenously. *J. Natl Cancer Inst.*, **83,** 1806–1812.

ROMANNI A, LIN JT, NIEDZWIECKI D, BUNNI M, PRIEST DG, BENTICO JR. (1991). Role of folypolyglutamates in biochemical modulation of fluoropyrimidines by leucovorin. *Cancer Res.*, **51,** 789–793.

SANTI DV, MCHENRY CS AND SOMMER M. (1974). Mechanisms of interaction of thymidylate synthase with 5-fluorodeoxyuridylate. *Biochemistry*, **12,** 471–480.

SOULE HD, VASQUEZ J, LONG AS AND BRENNAN M. (1973). A human cell line from a pleural effusion derived from a breast carcinoma. *J. Natl Cancer Inst.*, **51,** 1409–1416.

SPEARS CP, GUSTAVSON BG, MITCHELL MS, SPENCER D, BERNE M, BERSTEIN L AND DANENBERG PV. (1982). Thymidylate synthase inhibition in malignant tumors and normal liver. *Cancer Res.*, **44,** 4144–4150.

SWAIN SM, LIPPMAN ME, EGAN EF, DRAKE JC, STEINBERG SM AND ALLEGRA CJ. (1989). Fluorouracil and high-dose leucovrin in previously treated patients with metastatic breast cancer. *J. Clin. Oncol.*, **7,** 890–899.

TRAVE F, RUSTUM YM, PETRELLI NJ, HERRERA L, MITTLELMAN A, FRANK C AND CREANEN PJ. (1989). Plasma and tumor tissue pharmacology of high-dose intravenous leucovorin calcium in combination with fluorouracil in patients with advanced colorectal cancer. *J. Clin. Oncol.*, **6,** 1184–1191.

ULLMAN B, LEE M, MARTIN JF AND SANTI DV. (1978). Cytotoxicity of 5-fluoro-2'-deoxyuridine: requirement for reduced folate cofactors and antagonism by methotrexate. *Proc. Natl Acad. Sci. USA*, **75,** 980–983.

VALONE FH, FRIEDMAN MA, WITTLINGER PS, DRAKES T, EISENBERG PD, MALEC M, HANNIGAN JF AND BROWN JR BW. (1989). Treatment of patients with advanced colorectal carcinomas with fluorouracil alone, high-dose leucovorin plus fluorouracil, or sequential methotrexate, fluorouracil and leucovorin: a randomized trial of the Northern California Oncology Group. *J. Clin. Oncol.*, **7,** 1426–1436.

WOLMARK MN, ROCKETTE H, FISHER B, WICKERHAM DL, REDMOND C, FISHER ER, JONES J, MAMOUNAS EP, ORE L, PETRELLI NJ, SPURR CL, DIMITROV N, ROMOND EH, SUTHERLAND CM, KARDINAL CG, DEFUSCO A AND JOCHIMSEN P. (1993). The benefit of leucovorin-modulated fluorouracil as postoperative adjuvant therapy for primary colon cancer. Results from NSABP protocol C-03. *J. Clin. Oncol.*, **11,** 1879–1887.